# Systemic adalimumab induces peripheral corneal infiltrates: a case report

**DOI:** 10.1186/s12886-015-0047-6

**Published:** 2015-06-06

**Authors:** Alexandre Matet, Alejandra Daruich, Talal Beydoun, Jacques Cosnes, Jean-Louis Bourges

**Affiliations:** Department of Ophthalmology, Hotel-Dieu Hospital, AP-HP, and Sorbonne Paris Cité University, Paris Descartes faculty of medicine, 1 place du parvis Notre-Dame 75004, Paris, France; Department of ophthalmology, University of Lausanne, Jules-Gonin Eye Hospital, Fondation Asile des aveugles, Avenue de France 15, 1004, Lausanne, Switzerland; Department of Gastroenterology, Saint-Antoine Hospital, AP-HP, and Pierre et Marie Curie University, 184 rue du Faubourg Saint-Antoine 75012, Paris, France

**Keywords:** Cornea, Adalimumab, Tumor necrosis factor-alpha inhibitor, Adverse effects, Peripheral infiltrate, Crohn's disease

## Abstract

**Background:**

Tumor necrosis factor-alpha inhibitors are widely used agents in the treatment of immune disorders such as rheumatoid arthritis and inflammatory bowel disease. Despite their anti-inflammatory action, paradoxical drug-induced inflammatory events have been occasionally associated with the use of infliximab, etanercept, and in a lesser extent adalimumab. However, eye involvement is uncommon and anterior uveitis is the only reported ocular adverse manifestation. It can be induced by etanercept, but has also been described during adalimumab therapy. We present here the first report of recurrent peripheral corneal infiltrates following subcutaneous injections of adalimumab.

**Case presentation:**

A 34 year-old Caucasian woman with Crohn’s disease presented to the emergency department with bilateral red eyes and discomfort 36 hours after she received her bimonthly dose of subcutaneous adalimumab. Examination revealed bilateral peripheral corneal infiltrates with characteristic features of immune infiltrates. Symptoms and infiltrates regressed after topical corticosteroid therapy, but recurred after each adalimumab injection over the following weeks.

**Conclusion:**

Paradoxical immune reactions associated with tumor necrosis factor-alpha inhibitors may result either from hypersensitivity mechanisms, or from immune-complex deposition via anti-adalimumab antibodies. Both mechanisms could explain this newly described manifestation. Care should be taken to search for corneal infiltrates in the event of red eye symptoms during adalimumab therapy since they respond to topical corticosteroids and do not necessarily prompt the discontinuation of the immunosuppressive therapy.

**Electronic supplementary material:**

The online version of this article (doi:10.1186/s12886-015-0047-6) contains supplementary material, which is available to authorized users.

## Background

Adalimumab is a recombinant monoclonal antibody that inhibits tumor necrosis factor alpha (TNF-α), a pro-inflammatory cytokine. It is commonly employed for several immune-mediated disorders, including inflammatory bowel disease, ankylosing spondylitis and rheumatoid arthritis, with favorable safety reports [1, 2]. Yet, adverse events are progressively identified. Their diagnosis can be challenging since they often share features with the underlying inflammatory condition for which the drug is prescribed. The most common adverse manifestations include dermatitis, fever, interstitial pneumonia or vasculitis, but ocular involvement is very infrequent. To date, anterior uveitis is the only ocular adverse event registered in the literature [3]. In this report, we describe recurrent and bilateral peripheral corneal infiltrates caused by subcutaneous injections of adalimumab. To the best of our knowledge, this is the first report of adalimumab-induced corneal infiltrates.

## Case presentation

A 34 year-old Caucasian woman with Crohn’s disease presented to the eye emergency department at our institution with bilateral red eyes and discomfort. She had been wearing soft daily-wear contact lenses with monthly replacement schedule for the past 10 years. She had stopped wearing them 3 months before her visit as a consequence of fluctuating dry eye symptoms. She also reported a recent episode of interface dermatitis on her right ankle, confirmed by internal medicine specialists. She developed HLA-B27-negative ileal Crohn's disease at age 18, and required two intestinal resections at age 20 and 25 for stricturing disease. Thereafter, inflammation had been satisfactorily controlled by oral azathioprine. At the age of 30, 4 years before her visit to our emergency department, recurrence of clinical symptoms led to a switch from oral azathioprine to subcutaneous adalimumab. She had since been receiving 40 mg of subcutaneous adalimumab every 2 weeks.

Prior to the current episode, the patient had been evaluated biennially for 10 years by her attending ophthalmologist in the context of contact lens use. At each visit, she had been screened for ocular signs related to her inflammatory bowel disease. Her corneal status was unremarkable at all examinations. In particular, the patient did not have any history of meibomian gland disease or marginal keratitis.

**Fig. 1:**
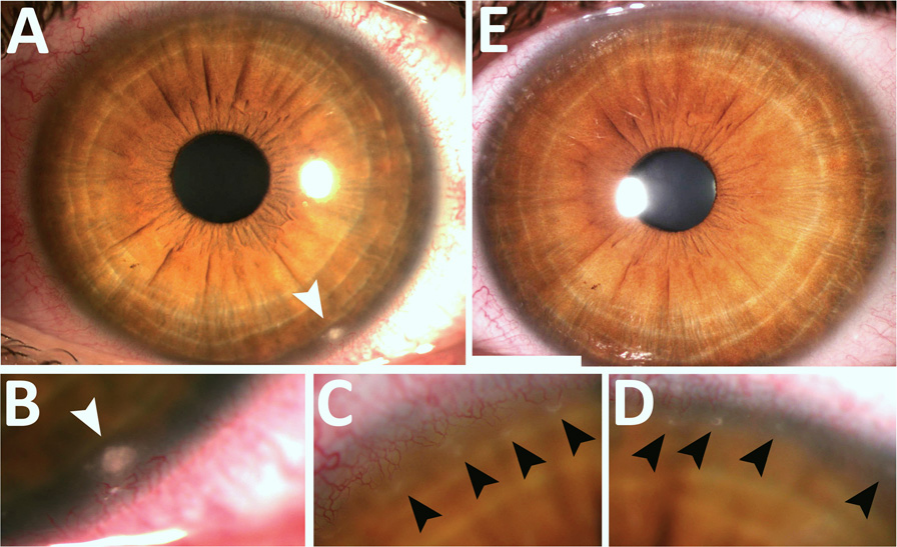
Peripheral corneal infiltrates secondary to systemic adalimumab for Crohn’s disease. **a**. Left eye of a 34 year-old female patient showing a grayish infiltrate near the inferior corneal margin (white arrow) observed 2 days after subcutaneous administration of adalimumab. **b**. Magnified view of the inferior infiltrate (white arrow) showing subtle corneal vascularization. **c** and **d**. String of smaller infiltrates along the superior corneal margin (black arrows). **e**. Regression of infiltrates after one week of topical dexamethasone T.I.D.

Ocular symptoms occurred 36 hours following the last adalimumab administration and were more intense in her left eye. The patient did not report any loss of vision. In addition to diffuse conjunctival hyperemia and peri-limbal injection, slit-lamp examination of her left eye revealed a white-grayish anterior stromal infiltrate near the inferior corneal margin, with a diameter of 0.3 mm (Fig. 1: a, b, white arrow), and a string of smaller lesions along the superior margin (Fig. 1: c, d, black arrows). We observed a single small lesion in her right eye, located along the superior nasal limbus. All signs shared characteristics of immune infiltrates: a hazy fluorescein stain with intact epithelium, a clear margin between infiltrate and limbus, and subtle corneal neovascularization. The anterior stromal localization of the lesions was visible on slit-lamp biomicroscopy (Additional file 1: Figure S1). Symptoms improved and infiltrates cleared with topical dexamethasone T.I.D (Fig. 1: e). Two weeks later, 24 hours after the next injection of adalimumab, the patient returned with recurrent symptoms. Clinical findings were identical to the first examination in both eyes and again disappeared with topical dexamethasone (Additional file 2: Figure S2 and Additional file 3: Figure S3). After a third episode that was managed in the same way, and at the patient’s request, sporadic ocular symptoms were considered acceptable with regard to the control of bowel inflammation, and adalimumab therapy was not discontinued. For the treatment of the few recurrences that occurred over the following months, dexamethasone was successfully replaced by rimexolone to reduce the risk of ocular hypertension. Two months after the first visit, trough serum adalimumab was 7.4 μg/mL, within therapeutic range (1.9 to 8.3 μg/mL) [4].

## Discussion

The features and timing of these recurrences strongly point to adalimumab injections as their causative factor. To the best of our knowledge, there is no previous report of peripheral corneal inflammation secondary to adalimumab.

The infiltrates could have been a manifestation of the inflammatory bowel disease itself. Ocular manifestations during Crohn’s disease indeed include scleritis, episcleritis, uveitis and less frequently peripheral corneal infiltrates, that may complicate sceritis [5, 6]. However, scleritis was absent in this case, and Crohn’s disease was quiescent. In fact, the residual serum level of adalimumab fell within therapeutic range [4], indicating efficient disease control.

Soft contact lens wear is unlikely to be the source of the peripheral infiltrates in this patient, since they had been discontinued 3 months before the onset of eye redness and discomfort. Intact corneal epithelium over the infiltrates and efficient control by steroids also rule out infectious keratitis. The absence of eyelid inflammation and the timing of recurrences do not support the hypotheses of meibomian gland disease, marginal keratitis or phlyctenulosis.

Ironically, TNF-α inhibitors are known to generate adverse inflammatory reactions despite their immunosuppressive action. Among them, adalimumab induces 3.5 % of hypersensitivity events, the lowest rate within this therapeutic class [7]. Cutaneous manifestations are the most frequent. They include type I hypersensitivity reactions at injection sites [8], paradoxical inflammation leading to “psoriasiform” lesions [9] and various types of dermatitis including interface dermatitis [10], as developed by our patient. Some reports also point to adalimumab as the causative agent in drug-induced interstitial pneumonia [11, 12], fever [13] or vasculitis [14, 15]. Yet, ocular adverse events have been rarely described following adalimumab, and the only reported manifestation is acute anterior uveitis [3].

Remarkably, in a recent United States-based registry analysis of uveitis induced by TNF-α inhibitors, adalimumab accounted for only 3 % of cases, far less than infliximab (24 %) and etanercept (73 %) [3]. Scleritis has also been described after etanercept [16], but not after adalimumab or infliximab. However, except for etanercept these agents are considered safe enough for the treatment of refractory inflammatory eye disease, including peripheral ulcerative keratitis [17], scleritis [18] and uveitis [19, 20].

Regarding its pathogenic mechanism, the formation of peripheral corneal infiltrates usually requires either immune-complex deposition or type III hypersensitivity [21]. In this patient, the presence of anti-adalimumab antibodies was very unlikely, since they would have been associated with low drug serum levels and treatment failure due to the neutralization of adalimumab molecules [22]. For this reason, laboratory testing was technically not feasible because the high residual adalimumab level would have neutralized any circulating anti-adalimumab antibodies.

## Conclusion

In this report, the patient developed symptomatic, recurrent corneal peripheral infiltrates after repeated subcutaneous administration of adalimumab, a TNF-α inhibitor. These infiltrates resulted either from an immune reaction to adalimumab, or from a paradoxical exacerbation of Crohn’s disease following adalimumab injections. Given the growing number of individuals under TNF-α inhibitor therapy, sterile corneal infiltrates should be meticulously looked for in the event of red eye symptoms. They respond to topical corticosteroid therapy without discontinuation of adalimumab.

## Consent

Written informed consent was obtained from the patient for publication of this Case report and any accompanying images. A copy of the written consent is available for review by the Editor of this journal.

## Additional files

Additional file 1: Figure S1. Localization of adalimumab-induced peripheral infiltrates in the anterior stroma. A. Magnified corneal photograph of the right eye showing a peripheral infiltrate near the superior nasal limbus and associated neovascularization (arrow). B. Magnified slit-lamp biomicroscopy showing the anterior stromal localization of the infiltrate (arrow). Additional file 2: Figure S2. Recurrence of the peripheral corneal infiltrates in the right eye following adalimumab subcutaneous injection. A. and B. Corneal photographs and magnified area of the superior nasal cornea after resolution of the first episode, showing a fine residual opacity and persistent neovascularization. C and D. Corneal photographs and magnified area of the same region after recurrence of symptoms 3 days after the next adalimumab injection, showing a recurrence of the peripheral infiltrate. Additional file 3: Figure S3. Recurrence of the peripheral corneal infiltrates in the left eye following adalimumab subcutaneous injection. A. Corneal photograph of the left eye, showing the absence of peripheral lesions after resolution of the first episode. B and C. Magnified areas in the superior and inferior temporal peri-limbal regions, after resolution of the first episode. D and E. Magnified areas of the same regions after a new onset of symptoms 2 days after the subsequent adalimumab subcutaneous injection, showing recurrent peripheral infiltrates (arrows).
