# Supplementary figures and images for: Systemic adalimumab induces peripheral corneal infiltrates: a case report
Source: BMC Ophthalmol. 2015 Jun 6;15:57. doi: 10.1186/s12886-015-0047-6 (PMC4456692; doi:10.1186/s12886-015-0047-6)

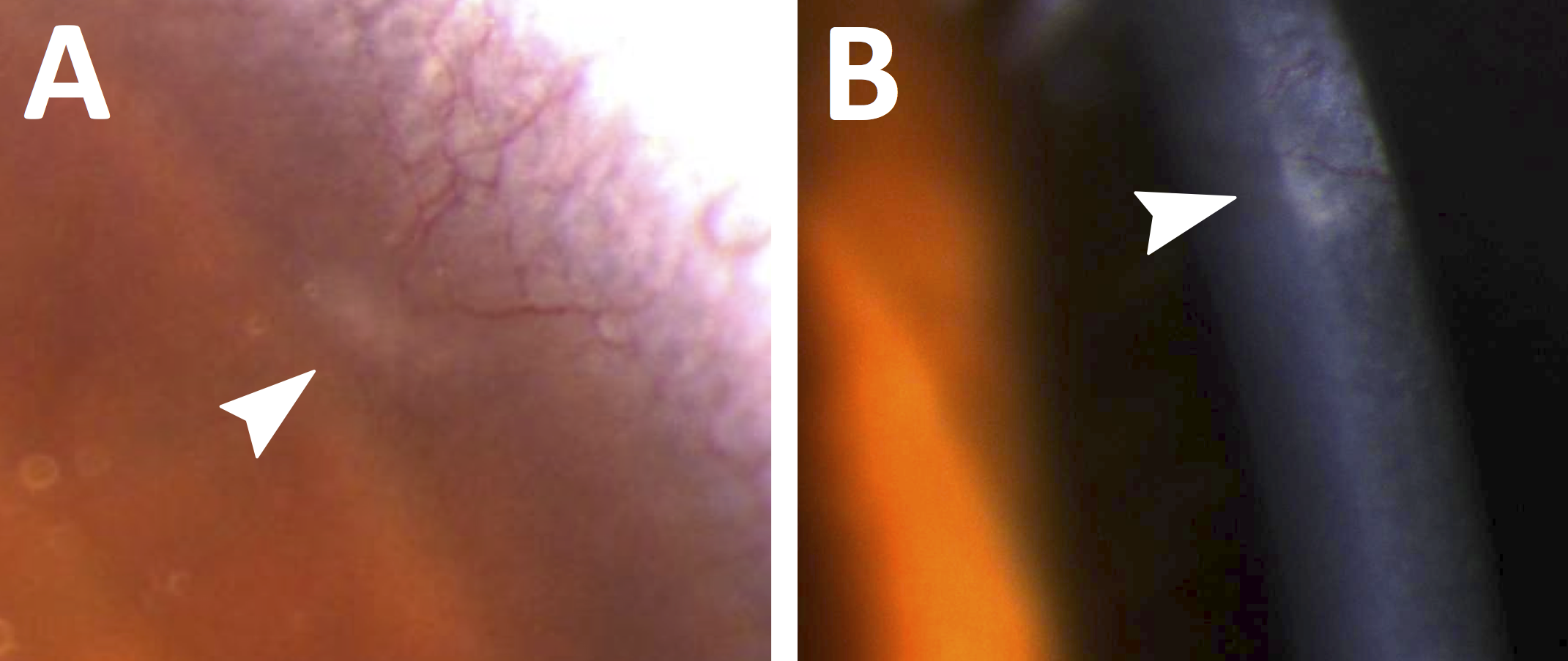

Supplement: Additional file 1: Figure S1. — Localization of adalimumab-induced peripheral infiltrates in the anterior stroma. A. Magnified corneal photograph of the right eye showing a peripheral infiltrate near the superior nasal limbus and associated neovascularization (arrow). B. Magnified slit-lamp biomicroscopy showing the anterior stromal localization of the infiltrate (arrow). [file 12886_2015_47_MOESM1_ESM.tiff]

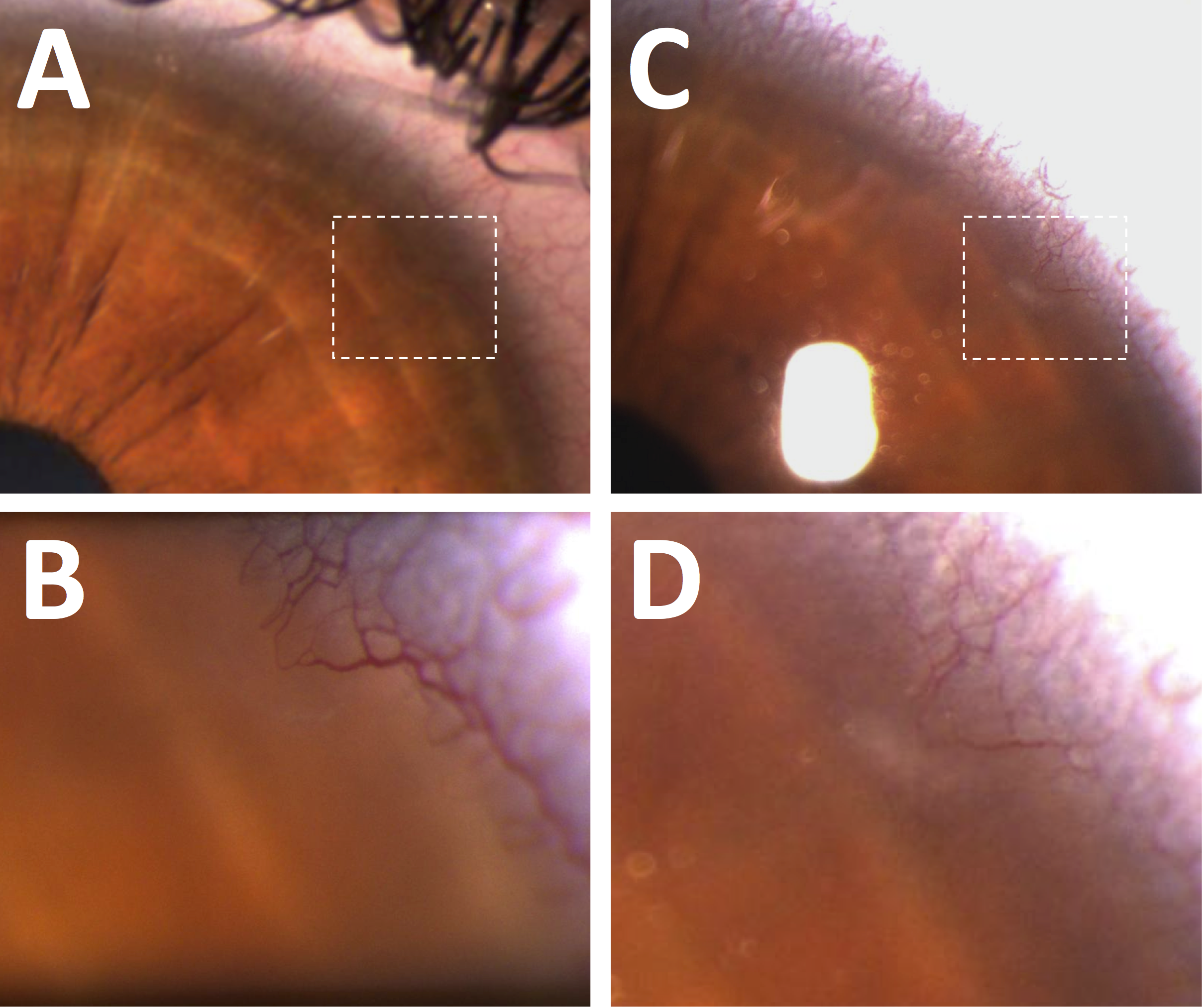

Supplement: Additional file 2: Figure S2. — Recurrence of the peripheral corneal infiltrates in the right eye following adalimumab subcutaneous injection. A. and B. Corneal photographs and magnified area of the superior nasal cornea after resolution of the first episode, showing a fine residual opacity and persistent neovascularization. C and D. Corneal photographs and magnified area of the same region after recurrence of symptoms 3 days after the next adalimumab injection, showing a recurrence of the peripheral infiltrate. [file 12886_2015_47_MOESM2_ESM.tiff]

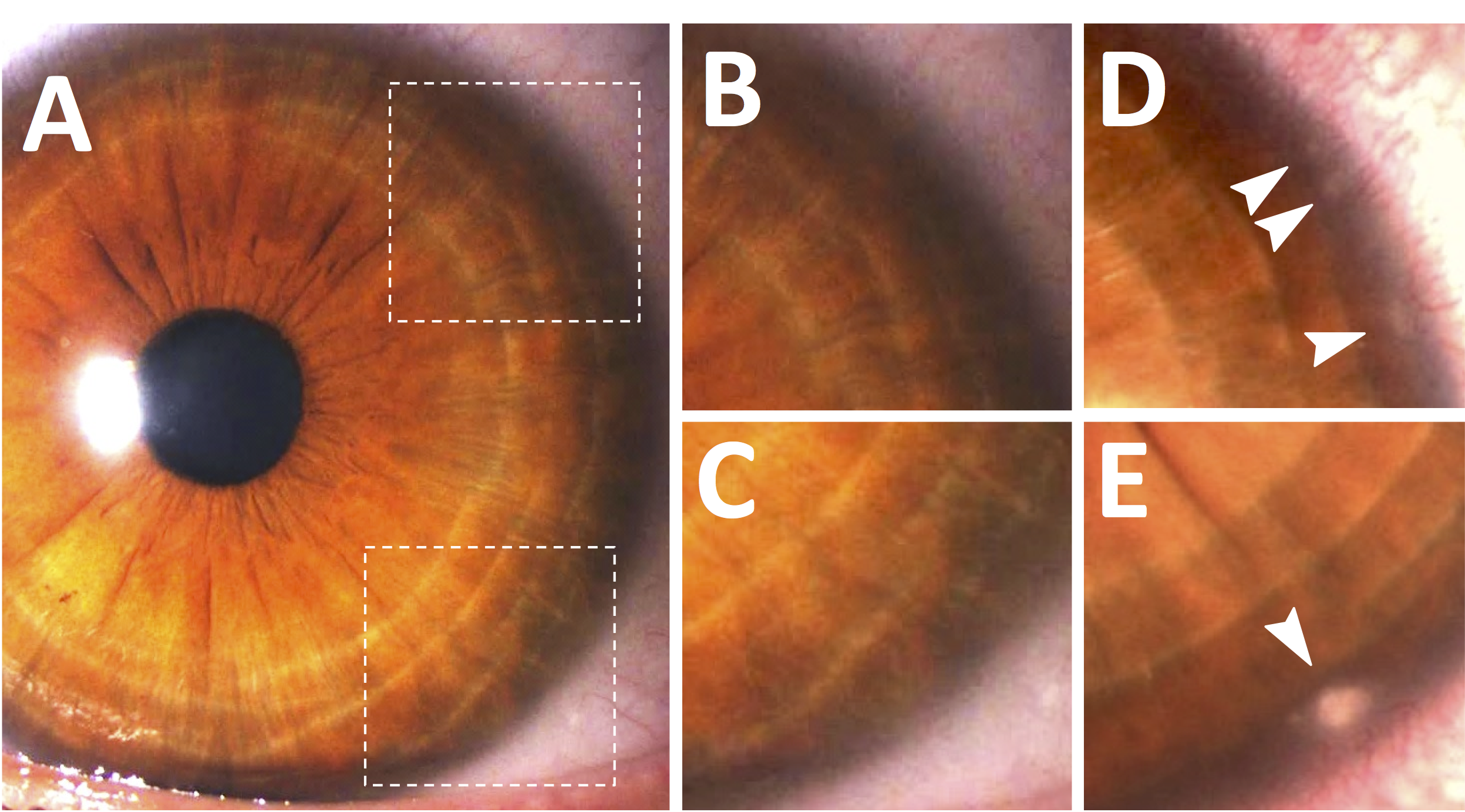

Supplement: Additional file 3: Figure S3. — Recurrence of the peripheral corneal infiltrates in the left eye following adalimumab subcutaneous injection. A. Corneal photograph of the left eye, showing the absence of peripheral lesions after resolution of the first episode. B and C. Magnified areas in the superior and inferior temporal peri-limbal regions, after resolution of the first episode. D and E. Magnified areas of the same regions after a new onset of symptoms 2 days after the subsequent adalimumab subcutaneous injection, showing recurrent peripheral infiltrates (arrows). [file 12886_2015_47_MOESM3_ESM.tiff]
